# Identification of cuproptosis-related biomarkers in dilated cardiomyopathy and potential therapeutic prediction of herbal medicines

**DOI:** 10.3389/fmolb.2023.1154920

**Published:** 2023-04-24

**Authors:** Rutao Bian, Yakuan Wang, Zishuang Li, Xuegong Xu

**Affiliations:** ^1^ Zhengzhou Hospital of Traditional Chinese Medicine, Zhengzhou, China; ^2^ Henan University of Chinese Medicine, Zhengzhou, China

**Keywords:** dilated cardiomyopathy, cuproptosis, ventricular remodeling, natural therapeutic compounds, immune infiltration

## Abstract

**Background:** Dilated cardiomyopathy (DCM) is one of the significant causes of heart failure, and the mechanisms of metabolic ventricular remodelling due to disturbances in energy metabolism are still poorly understood in cardiac pathology. Understanding the biological mechanisms of cuproptosis in DCM is critical for drug development.

**Methods:** The DCM datasets were downloaded from Gene Expression Omnibus, their relationships with cuproptosis-related genes (CRGs) and immune signatures were analyzed. LASSO, RF, and SVM-RFE machine learning algorithms were used to identify signature genes and the eXtreme Gradient Boosting (XGBoost) model was used to assess diagnostic efficacy. Molecular clusters of CRGs were identified, and immune Infiltration analysis was performed. The WGCNA algorithm was used to identify specific genes in different clusters. In addition, AUCell was used to analyse the cuproptosis scores of different cell types in the scRNA-seq dataset. Finally, herbal medicines were predicted from an online database, and molecular docking and molecular dynamics simulations were used to support the confirmation of the potential of the selected compounds.

**Results:** We identified dysregulated cuproptosis genes and activated immune responses between DCM and healthy controls. Two signature genes (FDX1, SLC31A1) were identified and performed well in an external validation dataset (AUC = 0.846). Two molecular clusters associated with cuproptosis were further defined in DCM, and immune infiltration analysis showed B-cell naive, Eosinophils, NK cells activated and T-cell CD4 memory resting is significant immune heterogeneity in the two clusters. AUCell analysis showed that cardiomyocytes had a high cuproposis score. In addition, 19 and 3 herbal species were predicted based on FDX1 and SLC31A1. Based on the molecular docking model, the natural compounds Rutin with FDX1 (-9.3 kcal/mol) and Polydatin with SLC31A1 (-5.5 kcal/mol) has high stability and molecular dynamics simulation studies further validated this structural stability.

**Conclusion:** Our study systematically illustrates the complex relationship between cuproptosis and the pathological features of DCM and identifies two signature genes (FDX1 and SLC31A1) and two natural compounds (Rutin and Polydatin). This may enhance our diagnosis of the disease and facilitate the development of clinical treatment strategies for DCM.

## 1 Introduction

Dilated cardiomyopathy (DCM) is a non-ischemic heart disease. It is characterized by unilateral or bilateral ventricular enlargement with systolic dysfunction. 70% of patients have heart failure as the primary clinical manifestation, which is progressively worsening (Conrad et al., 2019), with a 5-year survival rate of only 50%, and is the most common indication for heart transplantation (Gigli et al., 2019). The mechanistic factors that cause DCM are unknown and may be related to viral infections, autoimmune and genetic factors (Schultheiss et al., 2019). Unfortunately, satisfactory treatments are lacking due to DCM’s clinical heterogeneity and the pathological types’ complexity. Furthermore, DCM has no symptoms in its early stages and most patients lose their best chance of treatment after diagnosis. Current treatments focus on reducing cardiac energy requirements and preventing further deterioration of myocardial function (Walters et al., 2012). Therefore, there is a need to explore the pathological mechanisms of DCM in order to develop new therapeutic agents.

Metabolic defects and oxidative stress caused by disturbances in mitochondrial energy metabolism usually show signs of cardiac remodelling without overt disease. Mitochondrial energy metabolism involves the regulation of a large number of molecules, and Cu is an essential component of complex IV, which activates enzyme activity in the respiratory chain and in various biological processes, such as oxidative phosphorylation, aerobic respiration, and cell growth and development (Ruiz et al., 2021). High copper concentrations have been shown to be significantly associated with heart failure, including DCM and its prognosis (Huang et al., 2019), while copper chelation can repair mitochondria and improve cardiac function. Cuproptosis is a recently identified novel form of cell death (Tsvetkov et al., 2022). The main mechanisms of cuproposis is targeting lipid acylated tricarboxylic acid (TCA) cyclins to induce oligomerization of lipid acylated proteins and depletion of Fe-S cluster proteins, leading to proteotoxic stress and, ultimately, cell death (Chen L. et al., 2022; Tsvetkov et al., 2022). A recent study in diabetic cardiomyopathy confirmed the presence of cuproptosis in the pathological process (Huo et al., 2023). However, there are no studies on the role of cuproptosis in DCM, while disturbances in mitochondrial energy metabolism during DCM pathology have been investigated (Wu et al., 2021). Therefore, it is reasonable to speculate that a deeper understanding of the mechanisms of cuproptosis may help improve the treatment of DCM.

At this stage, specific drugs are lacking for inhibiting cuproptosis. Studies have primarily used copper ion chelators, and death can be somewhat mitigated. The use of natural medicinal herbs in the treatment of cardiovascular disease is gradually gaining acceptance, and have been used in the clinical management of DCM for many years. Studies have also confirmed that some herbal formulas (Sun et al., 2017; Peng et al., 2022) and natural compounds can inhibit apoptosis (Fan et al., 2022), improve mitochondrial energy metabolism (Duan et al., 2015) and inhibit myocardial fibrosis (Chen Q. et al., 2022). A study of herbs associated with cuproptosis showed that most natural compounds, such as resveratrol and quercetin, mainly depend on folic acid to regulate cuproptosis-related genes (CRGs) (Dayuan et al., 2023). However, herbal medicines and significant compounds that can regulate cuproposis remain unexplored. In the present study, we systematically examined differentially expressed CRGs and immune signatures between healthy and DCM individuals for the first time, screened for signature genes, and validated them. Finally, potential herbs and compounds were predicted by network pharmacology and further evaluated using molecular docking studies and molecular dynamics (MD) simulations. This study provides a theoretical reference and scientific basis for DCM mechanistic exploration and clinical treatment.

## 2 Materials and methods

### 2.1 Data resource

The DCM datasets GSE141910, GSE126569, GSE57338, GSE19303 and GSE21610 were downloaded from the Gene Expression Omnibus (GEO) database (https://www.ncbi.nlm.nih.gov/geo/). The GSE141910 dataset (GPL4372 platform) included 166 myocardial tissue samples from healthy controls and 166 DCM patients, and the GSE126569 dataset (GPL16791 and GPL20301 platform) included myocardial tissue samples from 18 healthy controls and 15 DCM samples, was selected and used as the exploration dataset. The GSE57338 dataset (GPL11532 platform) included myocardial tissue samples from 136 healthy controls and 82 DCM samples, and the GSE19303 dataset (GPL570 platform), including myocardial tissue samples from eight healthy controls and 40 DCM samples, and the GSE21610 dataset (GPL570 platform), including myocardial tissue samples from eight healthy controls and 21 DCM samples, were used for validation analyses. 13 genes (FDX1, LIPT1, LIAS, DLD, DBT, GCSH, DLST, DLAT, PDHA1, PDHB, SLC31A1, ATP7A, ATP7B) for CRGs were retrieved from previous publications (Huang et al., 2022; Lai et al., 2022) and used for subsequent analyses.

### 2.2 Differentially expressed genes (DEGs) screening

The “sva” package (Leek et al., 2012) combat algorithm was used to remove inter-batch differences in the GSE141910, GSE126569 datasets. The “limma” package (Ritchie et al., 2015) was used to identify DEGs between DCM and healthy controls. We extracted CRGs expression profiles from the total expression data and visualized the heat map using the “pheatmap” package. The packages “ggpubr” and “corrplot” were used for differential expression and CRGs correlation analysis. The Protein-Protein Interaction (PPI) network of 13 CRGs was constructed by the STRING database (https://cn.string-db.org/) (Szklarczyk et al., 2019).

### 2.3 Assessment of immune infiltration

The immune infiltration cells were further investigated by CIBERSORT(Zhao et al., 2023). Wilcoxon test was used to analyze the differences between the two groups, and Spearman correlation analysis was used to evaluate the correlation between CRGs and immune cells.

### 2.4 Identification of signature genes

The least absolute shrinkage and selection operator (LASSO) regression algorithm is a dimensionality reduction method that filters the unimportant variables by constraining the regression coefficients (λ) and finally obtains the results for the variable with the lowest prediction error, ‘glmnet " package (Chi et al., 2022) was used to construct the model. The support vector machine-recursive feature elimination (SVM-RFE) algorithm is an algorithm that ranks the scores of each feature gene by model training samples, and finally selects the desired feature gene by iteration. The model is built using the ‘e1071’ package (Lin et al., 2012), and the genes with the lowest cross-validation error are evaluated by setting k = 10 for screening. The Random Forest (RF) is a widely used machine learning algorithm based on decision tree theory that evaluates the key dimensions of the feature genes by bootstrap sampling of the training data, and finally ranks the importance of different predictor variables based on their predictive power.’ randomForest’ packages (Breiman et al., 2001) were used to construct the model with 168 trees as the best parameters for model classification. Overlapping genes were obtained by three machine learning methods with very high accuracy and were identified as the signature genes.

### 2.5 External validation of signature genes

To evaluate the accuracy of the screened signature genes and to perform diagnostic efficacy assessment as eXtreme Gradient Boosting (XGBoost) model trained eigenvalues by “XGBoost” packages (Ogunleye and Wang, 2020 **)**. First, we selected the DCM dataset (GSE141940) as the training set by the XGBoost model and applied an external dataset (GSE57338) for evaluation. The prognostic efficiency was evaluated by receiver operating characteristic (ROC), precision-recall (PR) curve, and area under the curve (AUC). Finally, two external DCM datasets, GSE19303 and GSE21610, were used for independent validation analysis to validate signature gene expression and to visualize ROC curves using the “pROC” package (Robin et al., 2011).

### 2.6 Unsupervised clustering of DCM patients

We performed unsupervised cluster analysis of DCM samples using the “ConsensuClusterPlus” package (Wilkerson and Hayes, 2010) to identify the different molecular clusters. The samples were grouped into clusters using a k-means calculus with 1,000 iterations. The maximum cumulative distribution function (CDF) index was chosen as the best k value (Wang et al., 2022). Principal component analysis (PCA) was used to visualize to determine if these genes could be used to differentiate samples, and CRGs expression differential analysis and immune infiltration were used to analyze the relationship between different clusters.

### 2.7 Weighted gene co-expression network analysis (WGCNA)

The “WGCNA” package (Langfelder and Horvath, 2008) is used to build a co-expression network, and R2 = 0.9 and the soft threshold β = 3 are selected. Then the genes are clustered into different modules, and the correlation between the modules and to find out the hub module associated with sample traits.

### 2.8 Functional enrichment analysis

The “ClusterProfiler” package was applied to perform Gene Ontology (GO) enrichment analysis with an *p* < 0.01 as a cut-off criterion. Finally, we performed a fast gene set enrichment analysis (FGSEA) (Korotkevich et al., 2021) using the hallmark gene set from the Molecular Signatures Database (MSigDB) as a feature gene to explore pathway differences between Clusters and the “pheatmap” package was used to visualize the results.

### 2.9 Analysis of scRNA-seq dataset

GSE109816 and GSE121893 were used to construct single-cell objects by using the “Seurat” package (Stuart et al., 2019) for analysis, setting each gene to be expressed in at least 3 cells, filtering out cells that expressed <200 or >6,000 genes in the pair samples. The “FindVariableFeatures” function was used to find highly variable genes. We then used the “harmony” package to filter the data by PCA and identified a total of 17 clusters. The “FindAllMarkers” function (min.pct = 0.25, logfc. threshold = 0.25) was used to identify marker genes and the representative cell types were annotated by the marker genes (Chaffin et al., 2022). Finally, AUCell analysis (Liu et al., 2022) was used to calculate the CGR scores in different cell types.

### 2.10 Key genes and herbal medicine prediction and molecular docking test

Based on the identified signature genes, we used the HERB database (http://herb.ac.cn/) (Fang et al., 2021) to back-predict the target herbs and components and selected herbs with regulatory effects on both signature genes for subsequent analysis. The 3D structures of the proteins encoded by the signature genes were downloaded from the Protein Data Bank (PDB, https://www.rcsb.org/), and the 3D structures of the compounds were downloaded from the PubChem database (https://pubchem.ncbi.nlm.nih.gov/). They were then imported into Chemdraw 3D, and the MM2 energy minimization module was used to obtain the most energy-efficient design and saved as a mol2 file. Protein structures were downloaded from the UniProt database (https://www.uniprot.org/), visualized separately by PyMOL 2.3.0 and Mgtools 1.5.6 to remove water molecules, add hydrogen, calculate the charge and merge non-polar hydrogen. Molecular docking models were performed using Autodock vina 1.1.2. The model with the lowest binding free energy was selected as the best docking model and visualized using PyMOL 2.3.0 and Discovery Studio 4.5.0.

### 2.11 Molecular dynamics simulation

Molecular dynamics simulations were carried out separately based on the small molecules and protein complexes obtained by docking as the initial structures, using AMBER 18 software (Salomon-Ferrer et al., 2013). Before the simulations, the charges of the small molecules were calculated using the antechamber module and Hartree-Fock (HF) SCF/6-31G* of the Gaussian 09 software (Frisch et al., 2009). The GAFF2 small molecule force field and the Ff14SB protein force field were used to describe them (Wang et al., 2004; Maier et al., 2015). The LEaP module was used to add hydrogen atoms to the system for each system, a truncated octahedral TIP3P solvent box (Mark and Nilsson, 2001)was added at a distance of 10 Å from the system, and Na^+^/Cl^−^ was added to the system for charge balance. Before the simulation, the design was energetically optimized by the steepest descent and the conjugate gradient methods. Subsequently, the plan was heated to 298.15 K using a 200 ps warming at a fixed volume and constant warming rate, and the system was heated to 298.15 K for a 500 ps NVT (isothermal isomeric) system simulation. The non-bond cut-off distance was set to 10 Å. The Particle mesh Ewald (PME) method (Sagui and Darden, 1999) was used to calculate the long-range electrostatic interaction, the SHAKE method (Kräutler et al., 2015) was used to limit the bond lengths of the hydrogen atoms, and the Langevin algorithm (Larini et al., 2007) was used for temperature control, where the collision frequency γ was set to 2 ps^−1^. The system pressure was 1 atm, the integration step was 2 fs, and the trajectories were saved at 10 ps intervals for subsequent analysis. Traces were saved at 10 ps intervals for subsequent analysis. The free energy of binding between protein and ligand was calculated by the MM/GBSA method for all systems (Rastelli et al., 2010; Genheden and Ryde, 2015), and can be calculated in the following useful way:

$${\Delta G}_{bind}={\Delta G}_{complex\;}–\;\left({\Delta G}_{receptor}+{\Delta G}_{ligand}\right)$$

$$={\Delta E}_{internal}+{\Delta E}_{VDW}+{\Delta E}_{elec}{+\Delta G}_{GB}+{\Delta G}_{SA}$$ (1)

In Equation 1, ${\Delta E}_{internal}$ is the internal energy, ${\Delta E}_{VDW}$ is the van der Waals energy and ${\Delta E}_{elec}$ is the electrostatic energy, G_GB_ is the electrostatic contribution to solvation and G_SA_ is the non-polar contribution to solvation.

## 3 Results

### 3.1 Dysregulation of cuproptosis and activation of the immune response in patients with DCM

**FIGURE 1:**
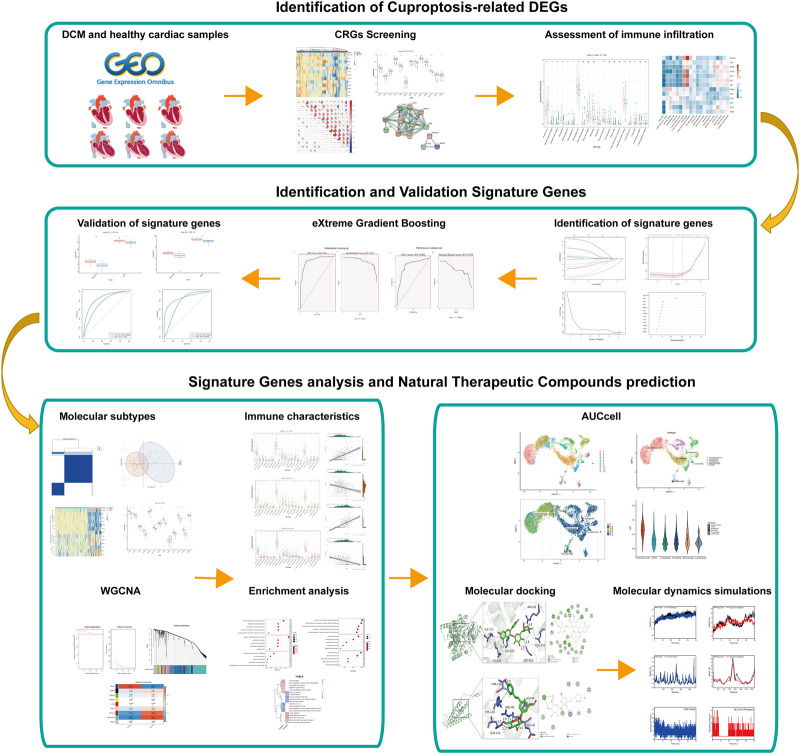
The flowchart of analysis process.

**FIGURE 2:**
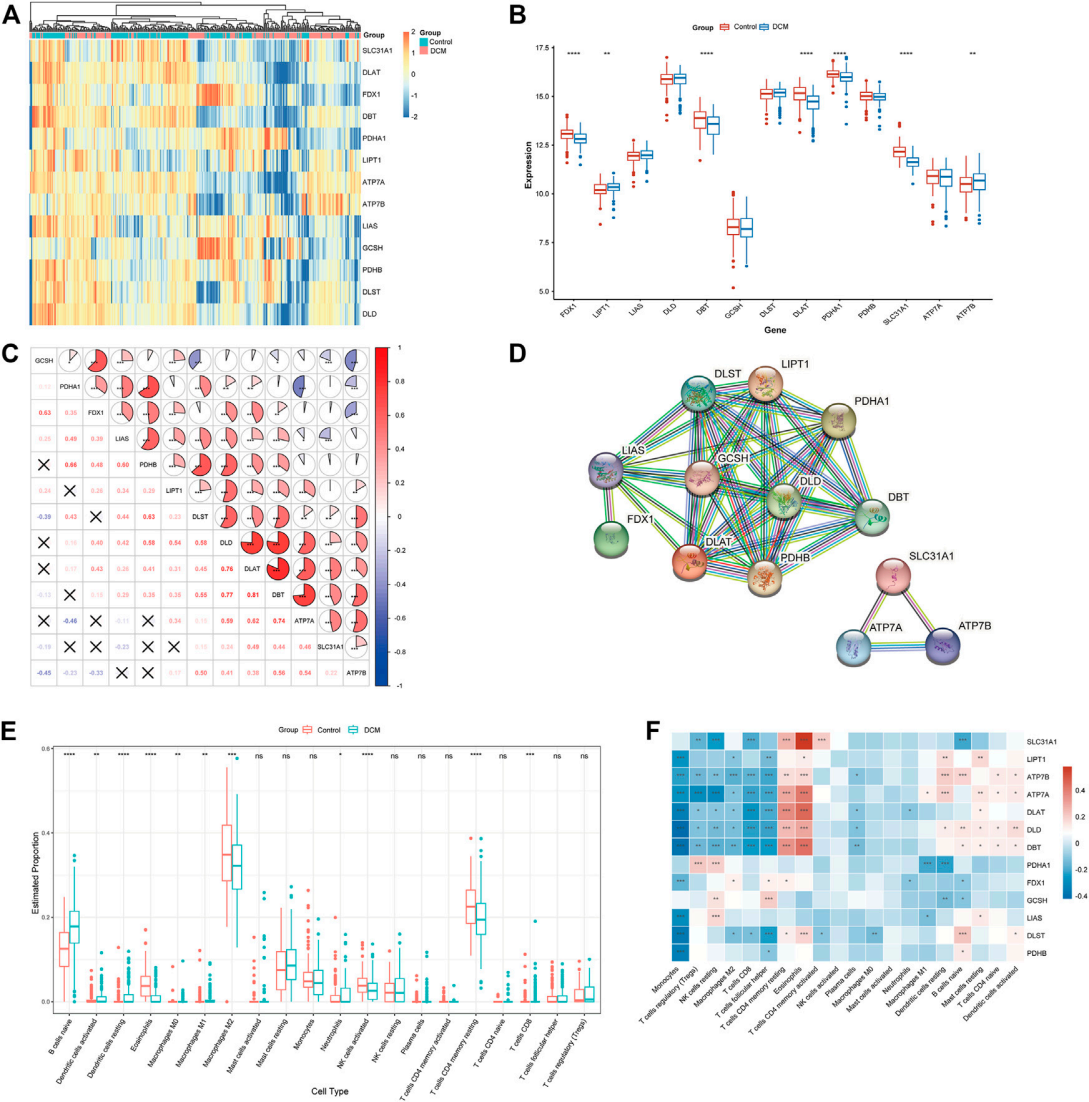
To clarify whether there were differences in the immune system between the Characterization of dysregulated CRGs in DCM. **(A)** Heat map showing the expression of 13 CRGs. **(B)** Boxplots showing the expression of the 13 CRGs between DCM and healthy controls. *p < 0.05, ***p < 0.001, ****p < 0.0001, ns, not significant. **(C)** Correlation analysis among the 13 CRGs. Red and blue represent positive and negative correlations. The area of the pie chart indicates the correlation coefficient. *p < 0.05, ***p < 0.001, ****p < 0.0001. **(D)** PPI network diagram of the CRGs. **(E)** Boxplots showing the difference in immune infiltration between DCM and healthy controls. *p < 0.05, ***p < 0.001, ****p < 0.0001, ns, not significant. **(F)** Correlation analysis between CRGs and infiltrating immune cells. Red and blue represent positive and negative correlations. *p < 0.05, ***p < 0.001, ****p < 0.0001.

The detailed flow of the study process is shown in Figure 1. The batch-to-batch differences were removed for the GSE141910 and GSE126569 datasets (Supplement Figure 1). The expression profiles of 13 CRGs between DCM and healthy controls using the combined data set (Figure 2A). Differential analysis revealed that FDX1, DBT, DLAT, PDHA1, and SLC31A1 were expressed at significantly higher levels in healthy controls samples than in DCM samples, while the opposite was for LIPT1 and ATP7B (Figure 2B). Subsequently, we performed a correlation analysis of the 13 CRGs (Figure 2C), and surprisingly some cuproptosis genes, such as DBT and DLAT, showed a strong synergistic effect (r = 0.81). In contrast, ATP7A and PDHA1 showed a significant antagonistic effect (r = -0.46). Further, PPI showed interactions between the CRGs (Figure 2D).

To clarify whether there were differences in the immune cell between DCM and healthy control, differences in the proportions of 22 immune cell types were assessed by the CIBERSORT algorithm (Figure 2E). The results showed that DCM patients exhibited higher levels of B-cell navie, Dendritic cells activated, Dendritic cells resting, Macrophages M0, Macrophages M1, Neutrophils, and T-cell CD8, suggesting that alterations in the immune system may be the main reason for the development of DCM. Meanwhile, correlation analysis between CRGs and immune cells (Figure 2F) showed a significant positive correlation between Eosinophils and SLC31A1 (r = 0.572), while a significant negative correlation between Monocytes and DLD (r = -0.426). These results suggest that CRGs may be vital in regulating DCM patients’ molecular and immune infiltration status.

### 3.2 Establishment and validation of signature genes

**FIGURE 3:**
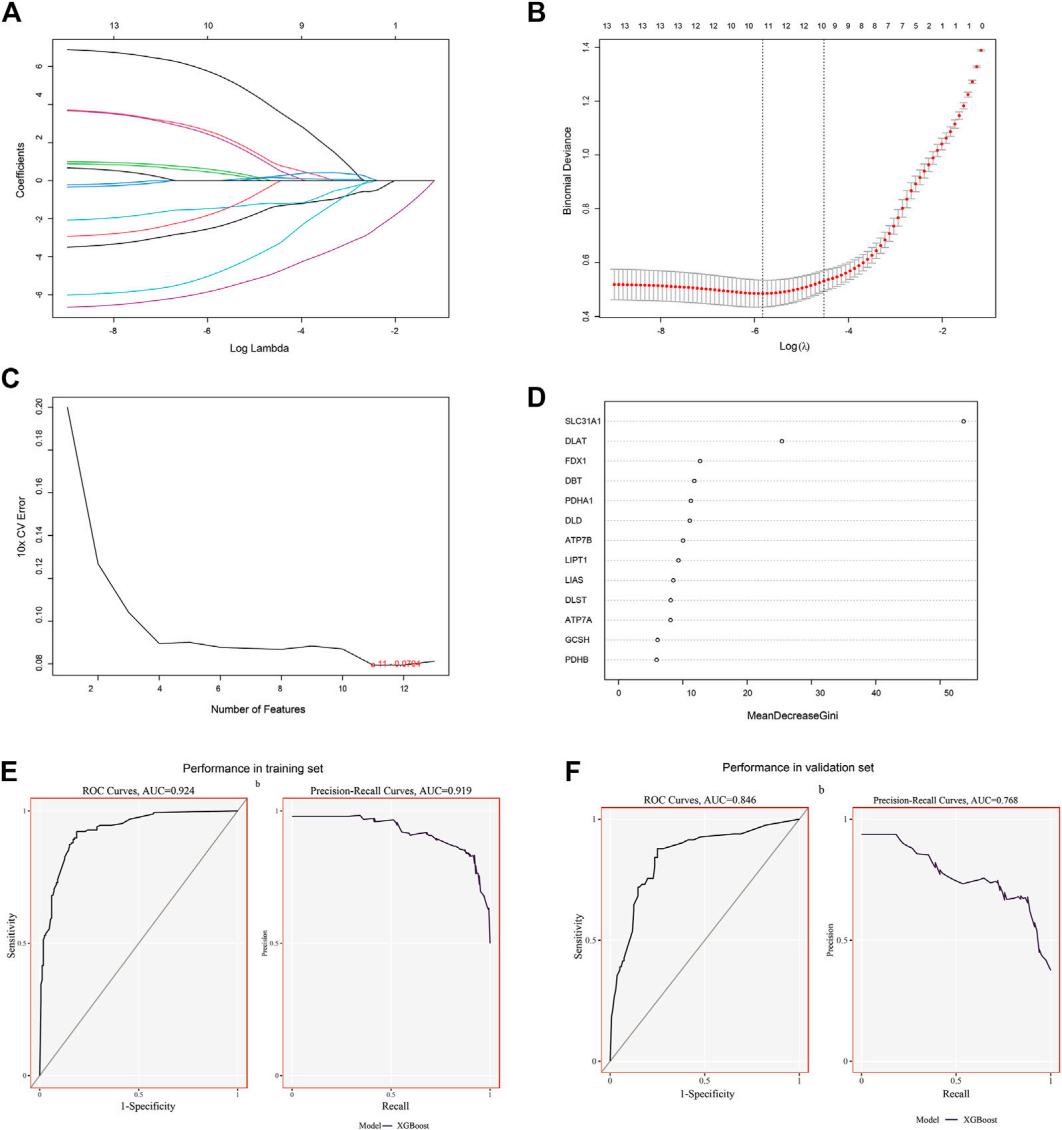
Identification and validation of signature genes. **(A)** Cross-validation for CRGs. **(B)** LASSO coefficient spectra of CRGs, and the λ value was confirmed as 0.010869. **(C)** The SVM-RFE algorithm was used to identify CRGs. **(D)** The RF algorithm was used to identify CRGs. **(E)** The performance of signature genes through the XGBoost model in the training set data of GSE141910 with AUC of 0.924 and PR of 0.919. **(F)** The performance of the signature genes by the XGBoost model in the validation data of GSE57338 with AUC of 0.846 and PR of 0.768.

The CRGs signature genes of DCM were identified by LASSO, RF and SVM-RFE algorithms. We ranked the genes using the LASSO algorithm (Figure 3A), and the top six genes were DLD, DLST, LIAS, PDHA1, SLC31A1, and FDX1. SVM-RFE is a supervised machine learning algorithm that ranks different features based on differences in predictive power (Figure 3B), and the top six genes were DLAT, DLD, SLC31A1, ATP7A, FDX1 and DBT. RF algorithm, the top six ranked genes were SLC31A1, DLAT, FDX1, DBT, PDHA1 and DLD (Figure 3D). The three algorithms found FDX1, SLC31A1 and DLD overlapping genes, but DLD was not statistically different in the dataset. Two signature genes (FDX1 and SLC31A1) were obtained.

**FIGURE 4:**
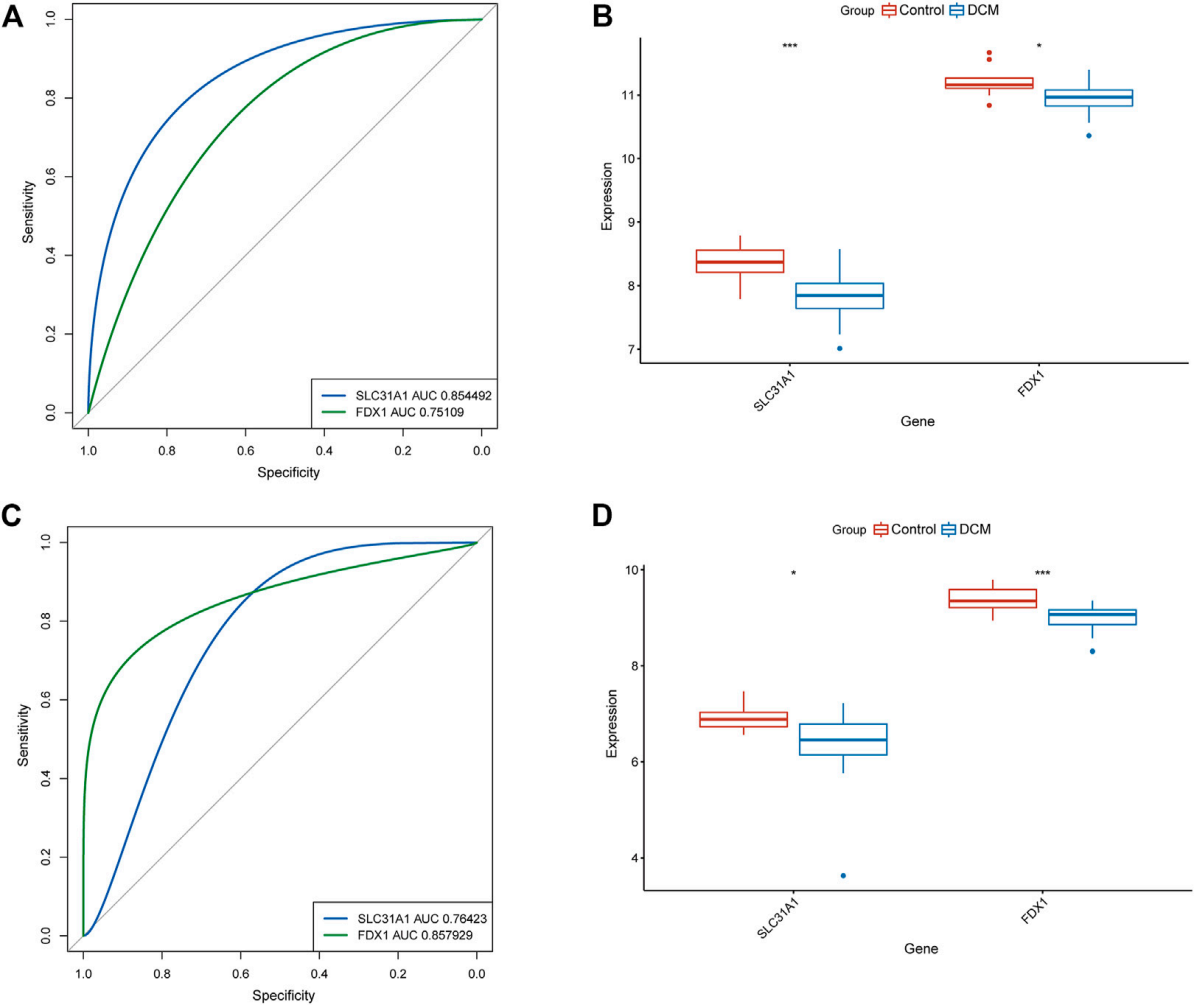
Validation of signature genes. **(A)** ROC curves showing the diagnostic performance of signature genes in GSE19303. **(B)** Validation of signature genes in GSE19303. * represents *p* < 0.05; ** represents *p* < 0.01; *** represents *p* < 0.001. **(C)** ROC curves showing the diagnostic performance of signature genes in GSE21610. **(D)** Validation of signature genes in GSE21610. * represents *p* < 0.05; ** represents *p* < 0.01; *** represents *p* < 0.001.

To determine the accuracy of our predicted signature genes, in the test dataset (GSE141910), the XGBoost model showed an AUC of 0.924 and PR of 0.919. In contrast, the validation dataset (GSE57338) showed an AUC of 0.846 and PR of 0.768, indicating that the signature genes prediction performance was significant. Two external datasets (GSE19303 and GSE21610) were used to test the significance of the signature genes. We found that in both datasets, FDX1 and SLC31A1 expression levels were significantly lower in the DCM group than in normal samples (Figures 4A,C). At the same time, the AUC for signature genes was more significant than 0.75 in both datasets, indicating good diagnostic efficacy (Figures 4B,D).

### 3.3 Identification of molecular features between clusters of cuproptosis in DCM

**FIGURE 5:**
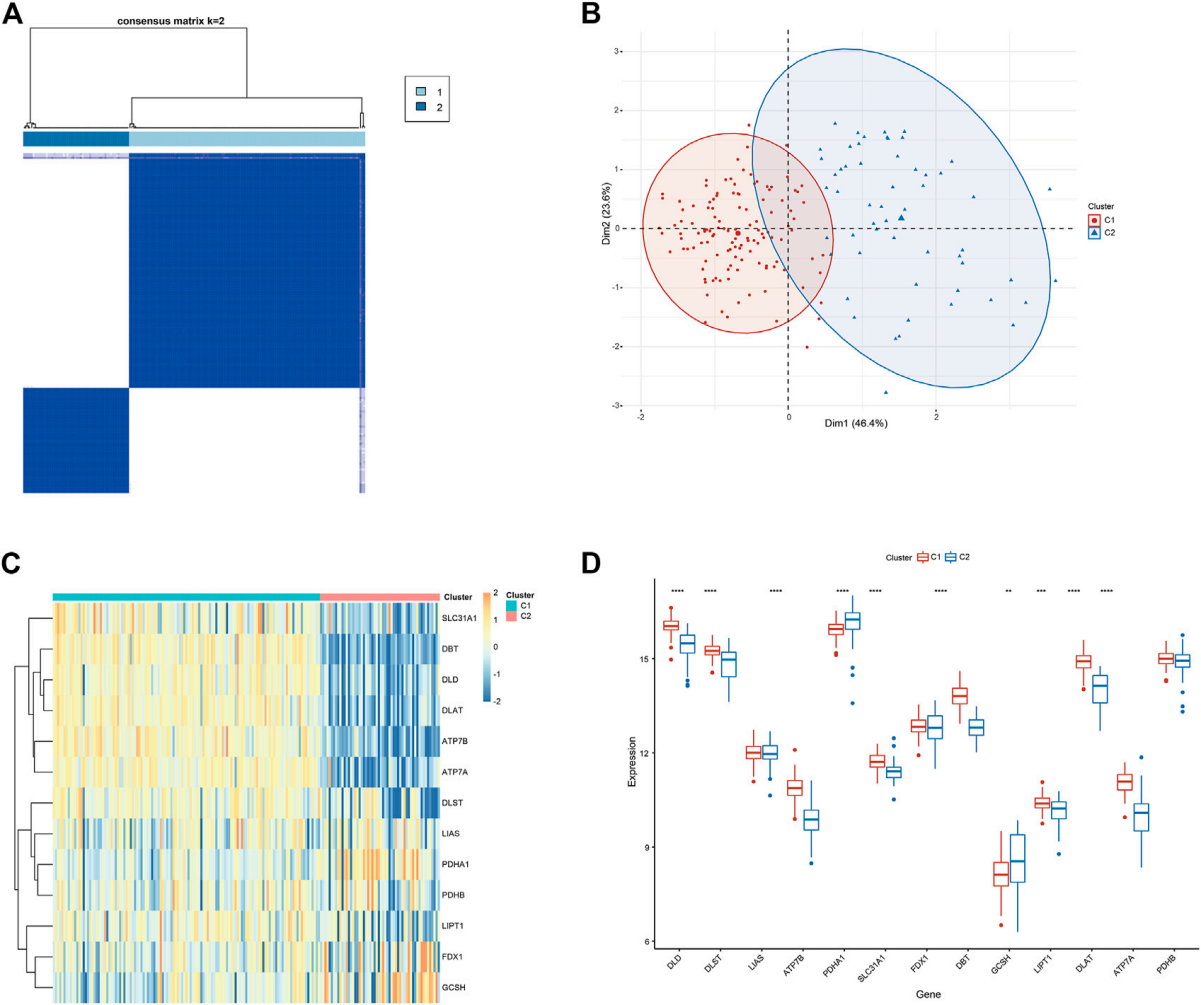
Identification of molecular clusters associated with cuproptosis in DCM. **(A)** Consensus clustering matrix at k = 2. **(B)** PCA visual analysis of two clustered distributions. **(C)** The heat map shows the expression of CRG between CRGcluster C1 and CRGcluster C2. **(D)** Box line plot showing the difference in expression between the CRGcluster C1 and CRGcluster C2. *p < 0.05, **p < 0.01 ***p < 0.001, ****p < 0.0001, ns, not significant.

To clarify the molecular clusters of cuproptosis in different populations of DCM, we grouped DCM samples based on the expression profiles of 13 CRGs using a consensus clustering algorithm. The number of clusters was most stable when the value of k was set to 2 (Figure 5A), and PCA analysis showed significant differences between these two clusters (Figure 5B). Combining the heat map of the consensus matrix, we finally divided the patients into two clusters, including CRGcluster C1 (n = 125) and CRGcluster C2 (n = 56), and the heat map showed significant differences between these two clusters (Figure 5C). We then examined the expression differences between CRGcluster C1 and CRGcluster C2, CRGcluster C1 showed high expression levels of DLD, DLST, ATP7B, SLC31A1, DBT, LIPT1, DLAT, and ATP7A, while Cluster 2 was characterized by enhanced expression of PDHA1 and GCSH (Figure 5D).

### 3.4 Immune infiltration characterization of CRGcluster

**FIGURE 6:**
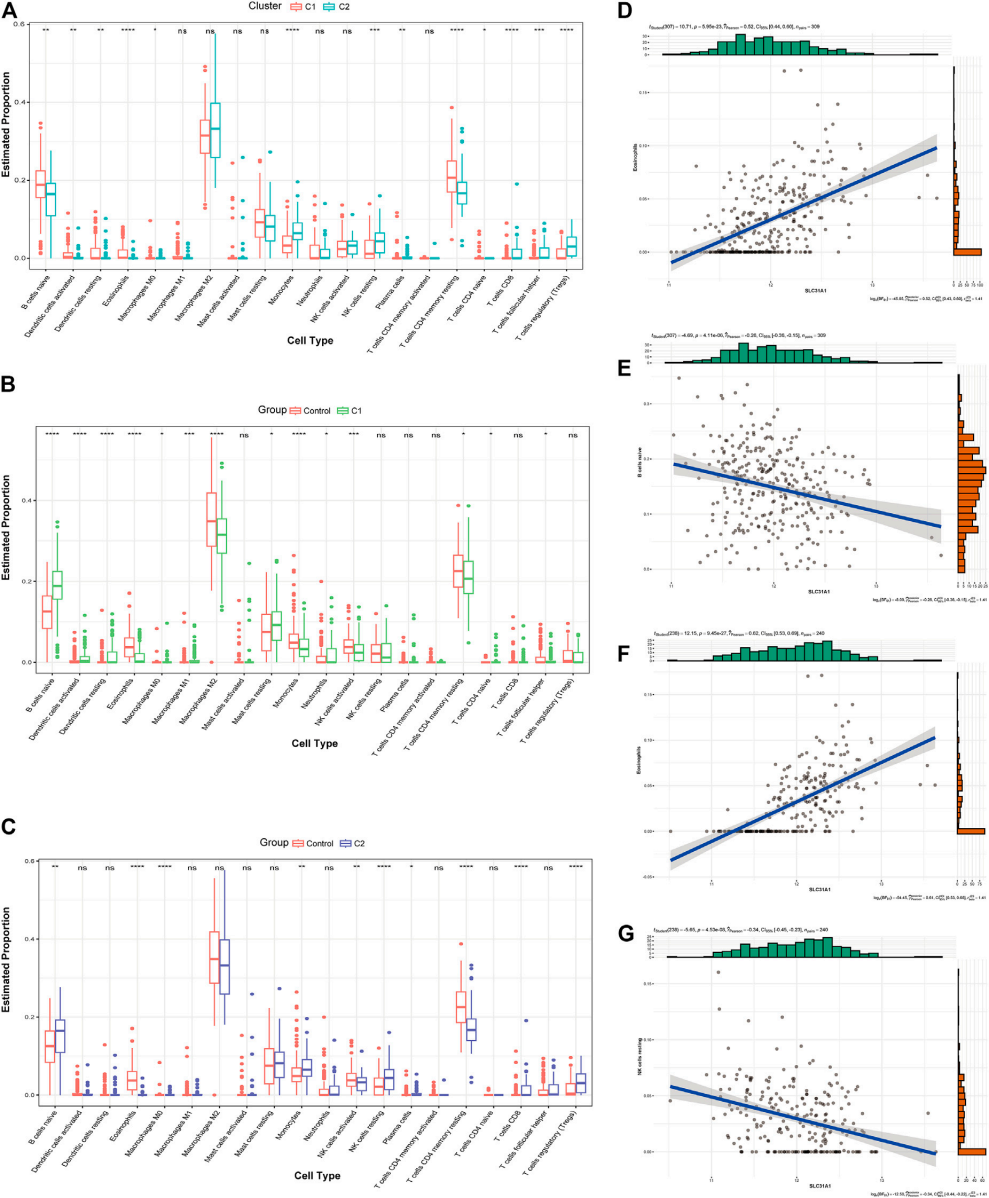
Analysis of the immunological profile between the two molecular clusters. **(A)** Box line plot of immune infiltration between two clusters. *p < 0.05, **p < 0.01 ***p < 0.001, ****p < 0.0001, ns, not significant. **(B)** Box line plot showing immune infiltration in CRGcluster C1 and healthy controls. *p < 0.05, **p < 0.01 ***p < 0.001, ****p < 0.0001, ns, not significant. **(C)** Box line plot showing immune infiltration in CRGcluster C2 and healthy controls. *p < 0.05, **p < 0.01 ***p < 0.001, ****p < 0.0001, ns, not significant. **(D, E)** Correlation analysis of immune cells with SLC31A1 in CRGcluster C1. **(F, G)** Correlation analysis of immune cells with SLC31A1 in CRGcluster C2.

We first assessed the immune infiltration between the two clusters. The results of the analysis CRGcluster C1 exhibited a higher proportion of B-cell naïve, Dendritic cells activated, Dendritic cells resting, Eosinophils, Macrophages M0, Plasma cells, T-cell CD4 memory resting, and T-cell CD4 naïve, while a large number of Monocytes, NK cells resting, T-cell CD8, T-cell follicular helper, and T-cell regulatory (Tregs) were more abundant in cluster 2 (Figure 6A). We then evaluated the immune infiltration between the two clusters and the healthy control samples (Figures 6B,C). The results showed that B-cell naive showed an increase in both clusters, but Eosinophils, NK cells activated and T-cell CD4 memory resting were decreased, and Macrophages M0 and Monocytes showed opposite status in both clusters. Based on the results of the immune infiltration analysis of both clusters, we also evaluated the correlation between the two signature genes and immune cells. In CRGcluster C1, SLC31A1 was positively correlated with Eosinophils (r = 0.52, Figure 6D), and SLC31A1 was negatively correlated with B-cell naive (r = -0.26, Figure 6E). In CRGcluster C2, SLC31A1 was positively correlated with Eosinophils (r = 0.62, Figure 6F), and SLC31A1 was negatively correlated with NK cells resting (r = -0.34, Figure 6G).

### 3.5 Co-expression module construction and enrichment analysis

**FIGURE 7:**
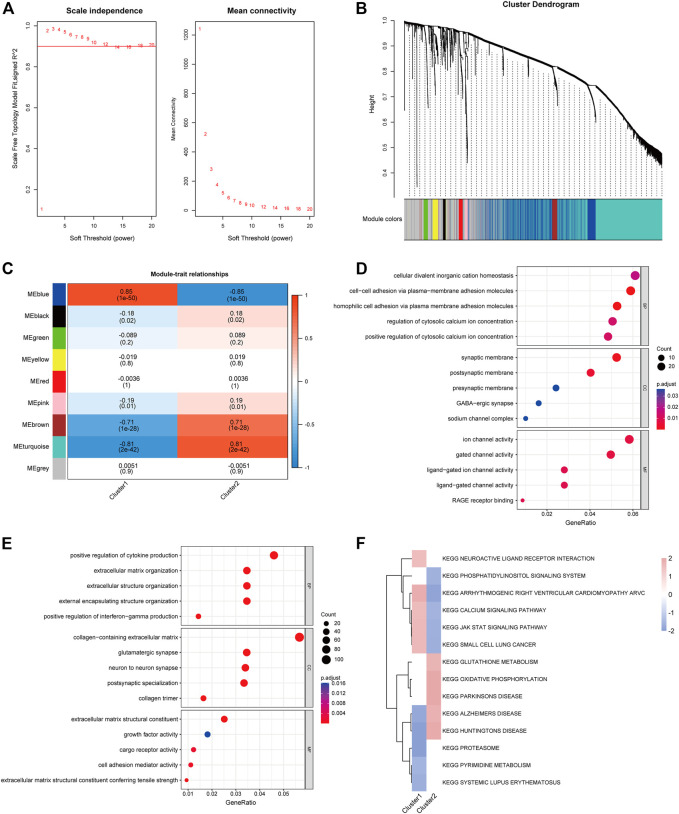
Co-expression pattern identification and enrichment analysis. **(A)** Selection of soft threshold power. **(B)** Tree diagram based on hierarchical clustering under optimal soft thresholds. **(C)** Heat map of correlations between nine modules and clinical features (CRGcluster C1, CRGcluster C2). **(D)** GO enrichment analysis of CRGcluster C1 MEblue module. **(E)** GO enrichment analysis of CRGcluster C2 turquoise module. **(F)** FGSEA enrichment analysis was performed on both clusters with a cut-off value of *p* < 0.01.

To identify differential genes in the two clusters, co-expression networks and modules were created between CRGcluster C1 and CRGcluster C2 using the WGCNA algorithm. Co-expressed gene modules were identified when the soft threshold was four, and the scale-free R2 was equal to 0.9 (Figure 7A). A total of nine different co-expression modules with different colours were obtained using the dynamic cut algorithm (Figure 7B). Analysis of module-clinical feature relationships showed that the MEblue module (1,149 genes) had the highest correlation with CRGcluster C1 (0.85) and the MEturquoise module (2,165 genes) had the highest correlation with CRGcluster C2 (0.81) (Figure 7C).

In addition, we performed GO enrichment analysis for both modules (Figures 7D,E). The enrichment results showed that in CRGcluster C1 (blue modules), they were mainly enriched in the regulation of cytosolic calcium ion concentration, synaptic membrane and ion channel activity, and the results of enrichment in CRGcluster C2 showed that in positive regulation of cytokine production, collagen-containing extracellular matrix, and extracellular matrix structural constituent (Supplementary Table S1). Finally, we performed FGSEA enrichment analysis relying on KEGG data from the “msigdbr” package to reveal differences in the molecular characteristics of the different clusters of vital pathways (Supplementary Table S2). Heatmaps show enrichment scores (NES) between clusters, neuroactive ligand receptor interaction, calcium signalling pathway, and JAK-STAT signalling pathway had higher NES in Cluster 1. In contrast, Cluster 2 showed glutathione metabolism, oxidative phosphorylation, and Parkinson’s disease were higher in NES.

### 3.6 Expression of CRGs in the scRNA-seq dataset

**FIGURE 8:**
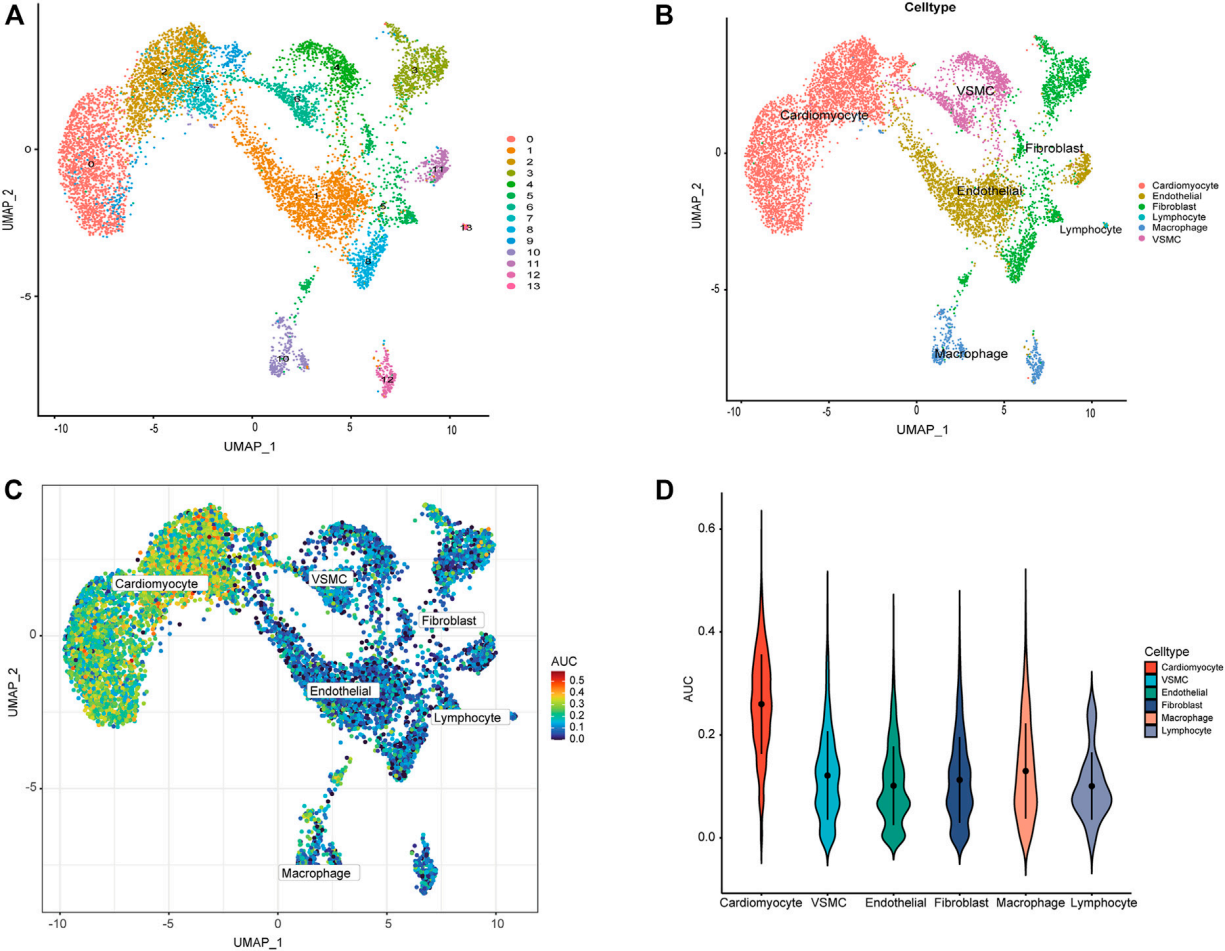
ScRNA-seq analysis reveals the expression of CRGs. **(A)** The tSNE plots of cardiac tissue cell populations. Each dot depicts a cell, depending on the cell population. **(B)** Defined cardiac tissue cells (cardiomyocytes, endothelial, fibroblasts, Lymphocyte, macrophages, and VSMC). **(C)** AUC of the CRG score visualized by T-SNE. **(D)** Visualization of CRG scores of different cells by violin plot.

We used the scRNA-seq dataset, which contained 14 healthy donors and 4 DCM patients. 9,138 cells passed standard quality control and were retained for subsequent analysis. We clustered all cells into 14 subgroups (Figure 8A), annotating cell populations according to previously published marker genes (Figure 8B), including cardiomyocytes, endothelial, fibroblasts, Lymphocyte, macrophages, and vascular smooth muscle cell (VSMC). The CRG activity of each cell was determined using the “AUCell” package. Higher AUC values were observed in cells expressing more genes, which were primarily cardiomyocytes (Figures 8C,D).

### 3.7 Molecular docking of key genes with herbal ingredients

We used the signature genes FDX1 and SLC31A1 as key genes for prediction, of which 19 herbs were found with FDX1 as the regulatory target, while three herbs were found with SLC31A1 as the regulatory target (Table 1). Surprisingly *Polygonum cuspidatum Sieb. Zucc. [P. reynoutria Makino; Reynoutria japonica Houtt.]* was predicted by both signature genes, and further exploration revealed that it contained 11 validated natural compounds.

**TABLE 1:** The predicted herbal medicines of signature genes.

Signature genes	Herbal medicines
FDX1	*Flos Pruni mume, Glycyrrhiza uralensis Fisch, Herba Fragariae, Polygonum cuspidatum Sieb.Zucc.[P.reynoutria Makino;Reynoutria japonica Houtt.], Extractum Piri Laiyanensis, Mangifera indica L.[M.austroyunnanensis Hu], Citric acid, Malus pumila Mill, Vitis vinifera, Crataegus pinnatifida Bge. var. major N.E.Br, Semen Pini Koraiensis, Pedicellus Melo, Pyrus communis, Helianthus annuus L, radix vitis romanetii, Crataegus cuneata, Hedychium forrestii, Crataegus scabrifolia, Semen Ziziphi Spinosae*
SLC31A1	*Trifolium fragiferum, Vinum, Polygonum cuspidatum Sieb.Zucc.[P.reynoutria Makino;Reynoutria japonica Houtt.].*

**FIGURE 9:**
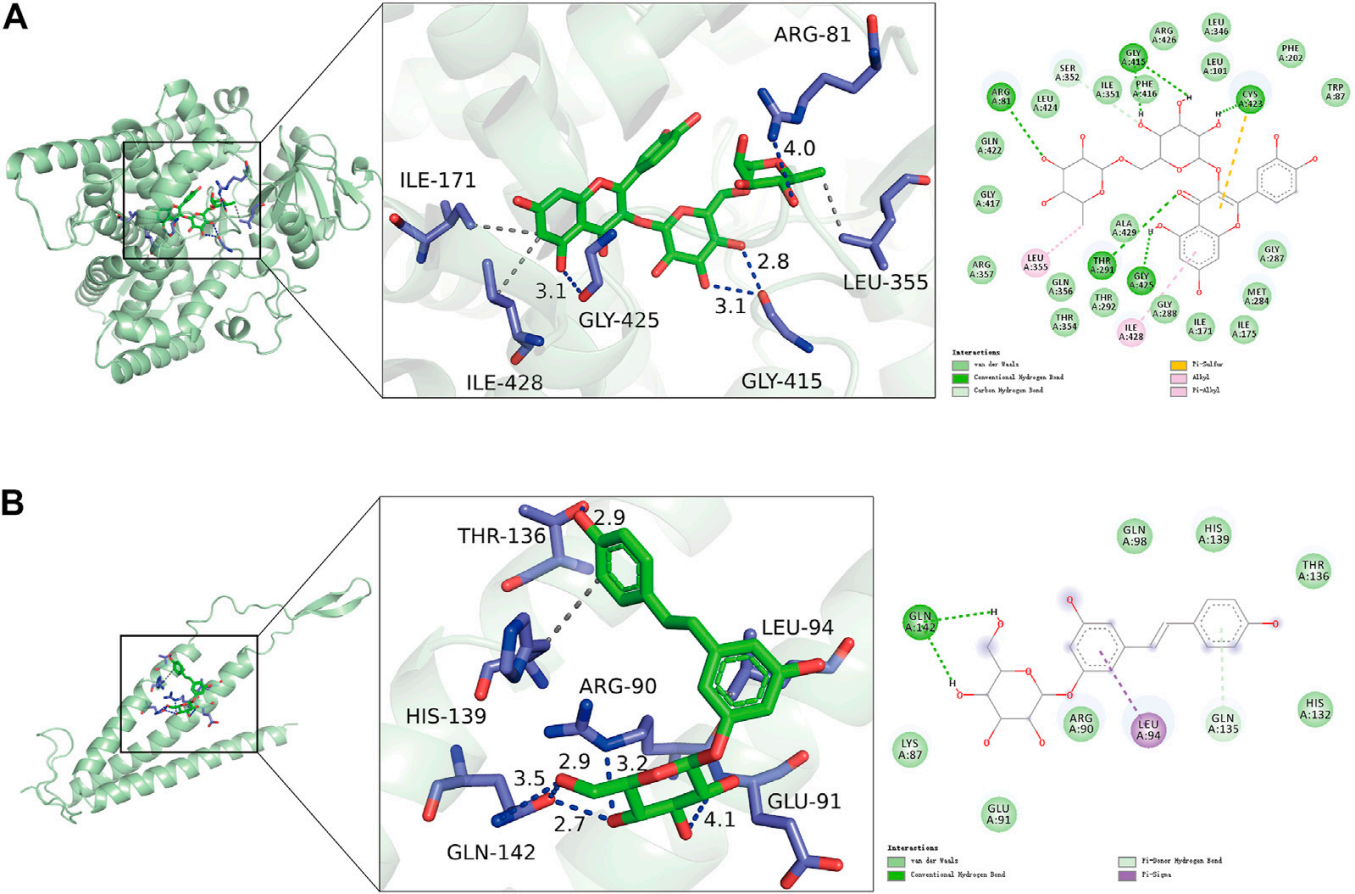
Molecular docking results of signature genes with related natural compounds. **(A)** Optimal binding model for Rutin to FDX1 with a minimum binding free energy of -9.3 kcal/mol. **(B)** Optimal binding model for Polydatin to SLC31A1 with a minimum binding free energy of -5.5 kcal/mol.

We further calculated the binding of FDX1 and SLC31A1 to 11 natural compounds separately and found good binding ability to a variety of compounds (Table 2). Typically, lower binding free energies lead to higher binding model stability, with binding free energies less than -5.0 kcal/mol indicating good binding activity between them (Hsin et al., 2013). The results showed molecular docking results showed that all compounds had negative binding energies. The Rutin docking well with FDX1 (Figure 9A) with a binding energy of -9.3 kcal/mol. Its binding energy was less than -7.0, indicating that the compound was able to spontaneously bind to the protein binding pocket with strong hydrogen bonding and hydrophobic interactions, while Polydatin bound to SLC31A1 (Figure 9B) with a binding energy of -5.5 kcal/mol and its binding energy was less than -5.0, indicating that the compound was able to interact well with the target protein.

**TABLE 2:** molecular docking analysis.

No.	Receptor name	Compound name	Energy (kcal/mol)
1	FDX1	Avicularin	-8.5
2	FDX1	Citricacid	-4.8
3	FDX1	Hyperin	-8.9
4	FDX1	Isoquercitrin	-8.5
5	FDX1	Malicacid	-4.1
6	FDX1	Polydatin	-8.8
7	FDX1	Quercitrin	-9.2
8	FDX1	Resveratrol	-8.1
9	FDX1	Rutin	-9.3
10	FDX1	Tartaricacid	-4.2
11	FDX1	Trans-Resveratrol	-8.1
12	SLC31A1	Avicularin	-4.7
13	SLC31A1	Citricacid	-3.3
14	SLC31A1	Hyperin	-4.8
15	SLC31A1	Isoquercitrin	-5.0
16	SLC31A1	Malicacid	-3.5
17	SLC31A1	Polydatin	-5.5
18	SLC31A1	Quercitrin	-4.7
19	SLC31A1	Resveratrol	-5.1
20	SLC31A1	Rutin	-4.8
21	SLC31A1	Tartaricacid	-3.9
22	SLC31A1	Trans-Resveratrol	-5.1

### 3.8 Molecular dynamic simulation of Rutin and Polydatin

**FIGURE 10:**
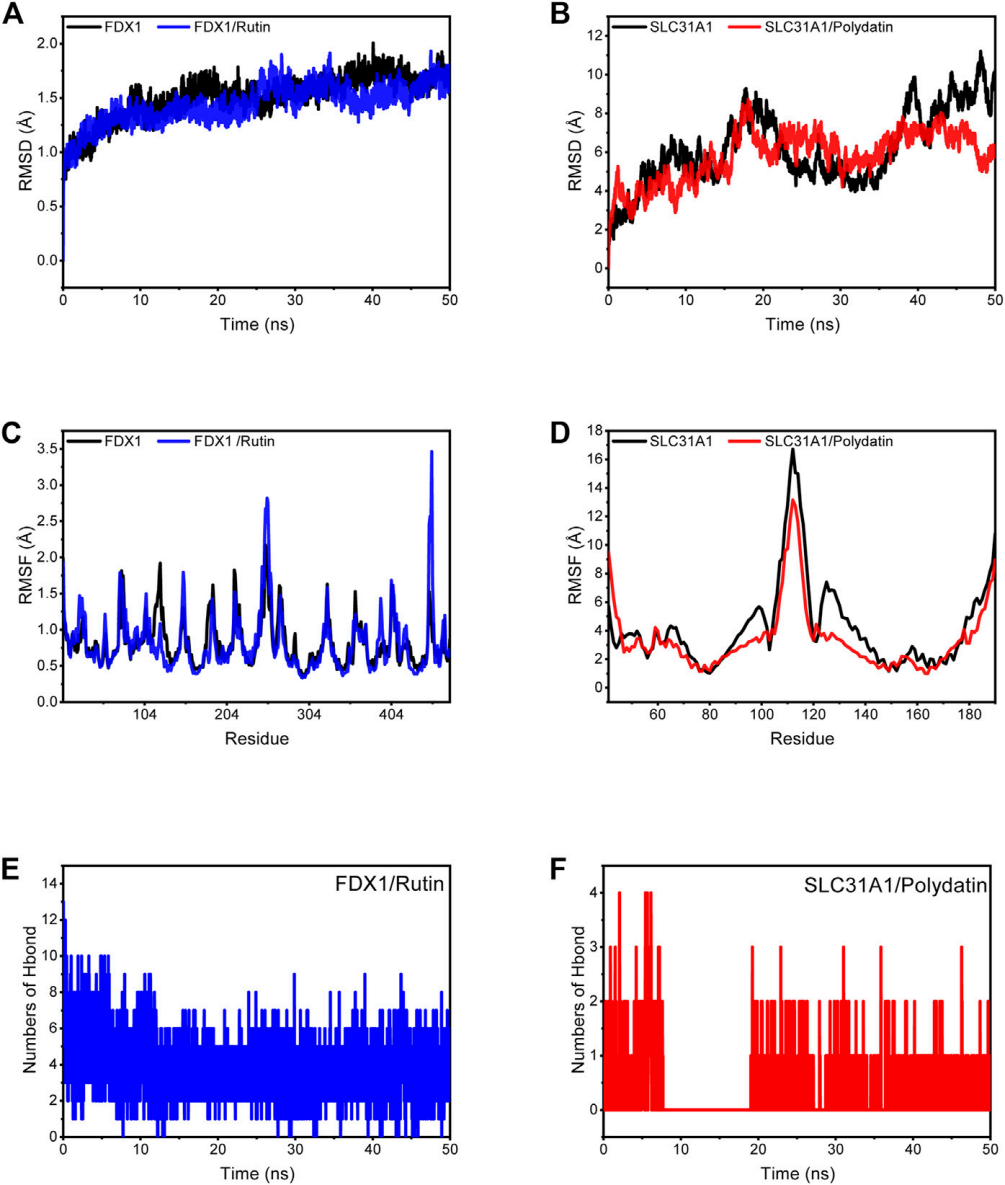
Molecular dynamics simulation. **(A)** RMSD plot showing FDX1, FDX1/Rutin along with time change. **(B)** RMSD plot showing SLC31A1, SLC31A1/Polydatin along with time change. **(C)** RMSF of FDX1 and FDX1/Rutin complex. **(D)** RMSF of SLC31A1 and SLC31A1/Polydatin complex. **(E)** Changes in the number of hydrogen bonds in the FDX1/Rutin. **(F)** Changes in the number of hydrogen bonds in the SLC31A1/Polydatin.

MD simulations were carried out to assess the flexibility and overall stability of the docked complexes. RMSD and RMSF plots were generated to determine the fluctuating behavior of the complexes. Without Rutin ligand binding to FDX1, the RMSD could converge rapidly and fluctuate. Still, the RMSD of the protein was lower than that of the empty protein when the small ligand molecule was bound, implying that the Rutin ligand allowed FDX1 to fluctuate stably (Figure 10A). In the SLC31A1/Polydatin complex, the RMSD of the empty protein and the complex fluctuates more, which is related to the long chain-like structure of the flexible protein system itself. Still, the RMSD of the complex fluctuates less than that of the empty protein, which means that SLC31A1 also shows a stable trend with the binding of small molecules (Figure 10B). These results suggest that the two complexes bind better. The RMSF can respond to the flexibility of the protein during molecular dynamics simulations. Usually, after the drug binds to the protein, the protein flexibility decreases, which in turn stabilizes the protein. As shown in Figure 10C and Figure 10D, we observed that in most regions, FDX1 and SLC31A1 showed lower RMSF under the influence of their respective ligands, especially SLC31A1 under the influence of Polydatin, which led to lower RMSF. Clearly, the compounds’ binding reduced the protein’s overall flexibility.

Based on the trajectory of molecular dynamics simulations, we calculated the binding energy using the MM/GBSA method, which can more accurately reflect the binding effect of small molecules and target proteins. As shown in Table 3, the binding energy of FDX1/Rutin was -37.16 ± 2.19 kcal/mol, while that of SLC31A1/Polydatin was -12.57 ± 1.18 kcal/mol. Negative values indicate that both molecules have binding affinity for the target protein, with lower values indicating stronger binding. Our calculations show that FDX1/Rutin and SLC31A1/Polydatin have a pooling effect, with FDX1/Rutin having a very strong binding affinity. By energy decomposition, we can see that the main contribution to the binding of FDX1/Rutin, SLC31A1/Polydatin is van der Waals energy, followed by electrostatic and non-polar solvation free energy. The number of hydrogen bonds formed by the FDX1/Rutin complex during the simulation ranged from 2-7 (Figure10E), with an enrichment of around 4 most of the time, implying that the complex formed more hydrogen bonds during the simulation and had a more substantial binding effect. For SLC31A1/Polydatin Figure 10F), we observed that the hydrogen bonds were more sparse, with no hydrogen bonding even sent during the simulation’s 7–19 ns phase, suggesting that hydrogen bonding is not the central role of this complex in maintaining binding.

**TABLE 3:** Binding free energies and energy components predicted by MM/GBSA (kcal/mol).

System name	FDX1/Rutin	SLC31A1/Polydatin
**Δ*E***_**vdw**_	-57.50 ± 1.98	-19.67 ± 1.79
**Δ*E***_**elec**_	-41.17 ± 3.86	-1.15 ± 3.28
**ΔG**_**GB**_	69.91 ± 6.94	10.94 ± 2.33
**ΔG**_**SA**_	-8.40 ± 0.16	-2.68 ± 0.35
**ΔG**_**bind**_	-37.16 ± 2.19	-12.57 ± 1.18

ΔE_vdW_:van der Waals energy.

ΔE_elec_:electrostatic energy.

ΔG_GB_: electrostatic contribution to solvation.

ΔG_SA_: non-polar contribution to solvation.

ΔG_bind_: binding free energy.

## 4 Discussion

Mitochondria are important organelles for copper ion storage and regulation, and the uptake, distribution and elimination of copper ions are tightly regulated. Dysregulation of copper homeostasis can produce cytotoxicity, leading to developing diseases such as Wilson′s disease (Bandmann et al., 2015) and Menkes disease (Bertini and Rosato, 2008). Cuproposis involves a novel type of cell death caused by toxic copper-dependent protein stress that has been demonstrated in tumor-related diseases, and many of copper chelation have been extensively studied and have bright prospects for development (Denoyer et al., 2015). However, the specific mechanisms of cuproposis and its regulatory role in various diseases have not been further investigated (Chen L. et al., 2022). This study comprehensively analysed the expression profiles of cuproposis regulators in healthy control and DCM patients. We found that CRGs expression was dysregulated in DCM patients, suggesting that CRGs plays an essential role in the development of the pathology. Subsequently, we calculated the correlation between CRGs, with DBT and DLAT showing a strong synergistic effect (r = 0.81) and ATP7A and PDHA1 showing a significant antagonistic effect (r = -0.46). After further screening for potential signature genes using LASSO, RF and SVM-RFE machine learning algorithms, we finally identified two signature genes (FDX1, SLC31A1). Testing of the external dataset GSE57338 by the XGBoost model showed that the signature genes had good diagnostic power for DCM-related cuprosis (AUC = 0.846), and the GSE19303 and GSE21610 datasets further validated that the signature genes were significantly different, which provided new insights for the diagnosis of DCM.

FDX1 is involved in fatty acid oxide (Schulz et al., 2022). Previous studies have shown that FDX1 is an upstream regulator of proteolipidation and a key regulator of cuproptosis in TCA (Dörsam and Fahrer, 2016). SLC31A1 is thought to be a high-affinity transporter protein for reduced Cu^+^ (Schweigel-Röntgen, 2014). It is primarily responsible for copper ions uptake, playing an important role in cellular copper homeostasis (Kim et al., 2010). Evidence suggests that SLC31A1 is required for copper transport to specific organs/tissues and that deficiency of SLC31A1 leads to abnormal cell growth (Lee et al., 2002). In contrast, the instability of Fe-S cluster proteins is closely related to FDX1. In addition, other studies have found that key genes related to Cu transport, such as SLC31A1 and ATP7B, may play an important role in regulating the development of cuproptosis. However, studies on FDX1 and SLC31A1 in DCM are lacking.

Advances in the treatment of DCM have been made in the past decades, and the identification of more appropriate molecular clusters is crucial to guide individualized treatment of DCM. We used unsupervised cluster analysis to illustrate the pattern of cuproposis regulation in DCM patients based on the expression profile of CRGs and identified two clusters. Subsequently, WGCNA analysis of the cluster feature modules and enrichment analysis showed that CRGcluster C1 was mainly enriched for the regulation of cell membrane calcium ion concentration. Synaptic membrane and ion channel activity, while CRGcluster C2 positively regulates cytokine production. FGSEA analysis revealed different enrichment pathways between CRGcluster C1 and CRGcluster C2, with neuroactive ligand receptor interaction, calcium signalling pathway and JAK-STAT signalling pathway having higher NES in cluster C1. In comparison, cluster C2 showed higher NES for glutathione metabolism, oxidative phosphorylation and Parkinson’s disease. Subsequently, we assessed the CRG activity of each cell using the scRNA-seq dataset, and higher AUC values were observed for cardiomyocytes.

In recent years as the pathological mechanisms of DCM have been investigated, the role of abnormal immune cells in ventricular remodelling has been revealed, and the role of immune-mediated inflammatory injury in the progression of DCM has been confirmed by several studies (Harding et al., 2023). Therefore, we analyzed the differences between the immune cells of DCM and healthy control Cardiac tissue. Analysis of the abundance of immune cells revealed that DCM patients exhibited higher levels of infiltration of B-cell navie, Dendritic cells activated, Dendritic cells resting, Macrophages M0, Macrophages M1, Neutrophils, and T-cell CD8. Correlation analysis of immune cells and CRGs revealed a significant positive correlation between Eosinophils and SLC31A1 (r = 0.572), while a significant negative correlation was found between DLD and Monocytes (r = -0.426). The role of different subpopulations of macrophages in DCM has been revealed by several studies (Kühl et al., 1995; Zhu et al., 2022), and they are not only involved in the development of apoptosis but also play an important role in the process of cardiac fibrosis (Cojan-Minzat et al., 2021). In addition, we also evaluated the immune infiltration between cluster C1 and cluster C2 *versus* normal samples. B-cell naive showed an increase in two clusters, while Eosinophils, NK cells activated and T-cell CD4 memory resting were decreased in two clusters. Further assessment of the correlation of the signature gene with immune cells in both clusters revealed a positive correlation between SLC31A1 and Eosinophils in both clusters. The Eosinophils (Zhang et al., 2022), NK cells activated (Kanda et al., 1992) and T-cell (Fang et al., 2022) in DCM have been confirmed by several studies, while the role of B-cell naive in DCM has not been investigated. This suggests that not only does cuproptosis play an important role in DCM immune inflammation, but that there are specific differences between Clusters.

The therapeutic role of herbal medicines in DCM has been confirmed by clinical studies (Zhu et al., 2016; Wang et al., 2021). In the present study, we identified a natural herbal medicine *Polygonum cuspidatum Sieb. Zucc. [P. reynoutria Makino; Reynoutria japonica Houtt.]* for the potential treatment of DCM, and 11 validated natural compounds were identified through HERB database. Molecular docking and molecular dynamics simulations were used to screen for targeting signature genes from 11 potential natural compounds. Rutin bound best to FDX1 with an energy of -9.3 kcal/mol, and Polydatin bound to SLC31A1 with an energy of -5.5 kcal/mol. MD simulations showed structural stability of both complexes, with pooling effects for FDX1/Rutin and SLC31A1/Polydatin, where FDX1/Rutin had a powerful binding affinity (-37.16 ± 2.19 kcal/mol). In contrast, the main contribution to binding was the van der Waals energy, followed by electrostatic energy and non-polar contribution to solvation. Several studies have confirmed the role of FDX1 and SLC31A1 in cuproposis, but drugs with therapeutic effects are lacking at this stage. Rutin is a herbal component that has also shown promising efficacy in treating several cardiovascular diseases. Rutin has antioxidant, anti-inflammatory, anti-apoptotic and improved energy metabolism properties and may play a therapeutic role in cardiovascular disease by reducing damage from toxic substances (Xianchu et al., 2018; Oluranti et al., 2021; Oluranti et al., 2022). Polydatin is therapeutic in cardiovascular disease by increasing superoxide dismutase (SOD) activity, inhibiting cardiomyocyte hypertrophy and regulating cellular calcium levels (Ding et al., 2014; Zhang et al., 2015; Yu et al., 2018). Rutin and Polydatins are the two compounds we found to have an inhibitory effect on cuproposis, primarily because they can bind tightly to FDX1 and SLC31A1. These results imply the potential of DCM drugs in inhibiting cuproposis.

This study is the first to systematically assess the role of cuproposis in DCM, identify signature genes and cell types, and predict potential natural compounds. However, some limitations need to be highlighted. First, our current study is based on retrospective data obtained from public databases and requires additional clinical or experimental evaluation for validation, and downstream mechanisms need further investigation. In addition, the potential correlation between CRG and immune response needs to be further explored.

## 5 Conclusion

In conclusion, we identified FDX1 and SLC31A1 as signature genes for cuproposis in DCM, and cardiomyocytes exhibiting higher cuproposis scores, B-cell naive, Eosinophils, NK cells activated and T-cell CD4 memory resting associated with immune infiltration of cuproposis in DCM, and identified potential natural compounds rutin and Polydatin. Our study advances the understanding of cuproposis in DCM and provides new insights into the role of mitochondria in its pathology.

## Figures and Tables

**Table:** 

**DCM**	dilated cardiomyopathy
**DEG**	differential expressed gene
**CRGs**	cuproptosis-related genes
**PPI**	protein-protein interaction
**LASSO**	least absolute shrinkage and selection operator
**RF**	random forest
**SVM-RFE**	support vector machine-recursive feature elimination
**XGBoost**	eXtreme Gradient Boosting
**CDF**	cumulative distribution function
**ROC**	receiver operating characteristic curve
**AUC**	area under curve
**PR**	precision-recall
**WGCNA**	Weighted gene co-expression network analysis
**GO**	Gene Ontology
**KEGG**	Kyoto Encyclopedia of Genes and Genomes
**MSigDB**	Molecular Signatures Database
**FGSEA**	Fast gene set enrichment analysis
**NES**	enrichment scores
**GEO**	Gene Expression Omnibus
**MD**	molecular dynamics
**PCA**	principal component analysis

## Data Availability

The datasets presented in this study can be found in online repositories. The names of the repository/repositories and accession number(s) can be found in the article/Supplementary Material.

## References

[B1] Bandmann O. Weiss K. H. Kaler S. G. (2015). Wilson's disease and other neurological copper disorders. Lancet Neurol. 14 (1), 103–113. 10.1016/s1474-4422(14)70190-5 25496901 PMC4336199

[B2] Bertini I. Rosato A. (2008). Menkes disease. Cell Mol. Life Sci. 65 (1), 89–91. 10.1007/s00018-007-7439-6 17989919 PMC11131655

[B3] Breiman L. Breiman L. Cutler R. A. (2001). Random forests machine learning. J. Clin. Microbiol. 2, 199–228.

[B4] Chaffin M. Papangeli I. Simonson B. Akkad A. D. Hill M. C. Arduini A. (2022). Single-nucleus profiling of human dilated and hypertrophic cardiomyopathy. Nature 608 (7921), 174–180. 10.1038/s41586-022-04817-8 35732739 PMC12591363

[B5] Chen L. Min J. Wang F. (2022a). Copper homeostasis and cuproptosis in health and disease. Signal Transduct. Target Ther. 7 (1), 378. 10.1038/s41392-022-01229-y 36414625 PMC9681860

[B6] Chen Q. Zeng Y. Yang X. Wu Y. Zhang S. Huang S. (2022b). Resveratrol ameliorates myocardial fibrosis by regulating Sirt1/Smad3 deacetylation pathway in rat model with dilated cardiomyopathy. BMC Cardiovasc Disord. 22 (1), 17. 10.1186/s12872-021-02401-y 35081907 PMC8793224

[B7] Chi H. Xie X. Yan Y. Peng G. Strohmer D. F. Lai G. (2022). Natural killer cell-related prognosis signature characterizes immune landscape and predicts prognosis of HNSCC. Front. Immunol. 13, 1018685. 10.3389/fimmu.2022.1018685 36263048 PMC9575041

[B8] Cojan-Minzat B. O. Zlibut A. Agoston-Coldea L. (2021). Non-ischemic dilated cardiomyopathy and cardiac fibrosis. Heart Fail Rev. 26 (5), 1081–1101. 10.1007/s10741-020-09940-0 32170530

[B9] Conrad N. Judge A. Canoy D. Tran J. Pinho-Gomes A. C. Millett E. R. C. (2019). Temporal trends and patterns in mortality after incident heart failure: A longitudinal analysis of 86 000 individuals. JAMA Cardiol. 4 (11), 1102–1111. 10.1001/jamacardio.2019.3593 31479100 PMC6724155

[B10] Dayuan Z. Lan L. Hui C. Huanjie L. Yumei L. Yumiao L. (2023). Study on computer screening and drug properties of herbs intervening in copper death. Comput. Math. Methods Med. 2023, 3311834. 10.1155/2023/3311834 36684691 PMC9848818

[B11] Denoyer D. Masaldan S. La Fontaine S. Cater M. A. (2015). Targeting copper in cancer therapy: 'Copper that cancer. Metallomics 7 (11), 1459–1476. 10.1039/c5mt00149h 26313539

[B12] Ding W. Dong M. Deng J. Yan D. Liu Y. Xu T. (2014). Polydatin attenuates cardiac hypertrophy through modulation of cardiac Ca2+ handling and calcineurin-NFAT signaling pathway. Am. J. Physiol. Heart Circ. Physiol. 307 (5), H792–H802. 10.1152/ajpheart.00017.2014 25015961

[B13] Dörsam B. Fahrer J. (2016). The disulfide compound α-lipoic acid and its derivatives: A novel class of anticancer agents targeting mitochondria. Cancer Lett. 371 (1), 12–19. 10.1016/j.canlet.2015.11.019 26604131

[B14] Duan Z. Z. Li Y. H. Li Y. Y. Fan G. W. Chang Y. X. Yu B. (2015). Danhong injection protects cardiomyocytes against hypoxia/reoxygenation- and H2O2-induced injury by inhibiting mitochondrial permeability transition pore opening. J. Ethnopharmacol. 175, 617–625. 10.1016/j.jep.2015.08.033 26320687

[B15] Fan S. Hu Y. You Y. Xue W. Chai R. Zhang X. (2022). Role of resveratrol in inhibiting pathological cardiac remodeling. Front. Pharmacol. 13, 924473. 10.3389/fphar.2022.924473 36120366 PMC9475218

[B16] Fang C. Lv Z. Yu Z. Wang K. Xu C. Li Y. (2022). Exploration of dilated cardiomyopathy for biomarkers and immune microenvironment: Evidence from RNA-seq. BMC Cardiovasc Disord. 22 (1), 320. 10.1186/s12872-022-02759-7 35850644 PMC9290235

[B17] Fang S. Dong L. Liu L. Guo J. Zhao L. Zhang J. (2021). Herb: A high-throughput experiment- and reference-guided database of traditional Chinese medicine. Nucleic Acids Res. 49 (D1), D1197–d1206. 10.1093/nar/gkaa1063 33264402 PMC7779036

[B18] Frisch M. Trucks G. Schlegel H. Scuseria G. Robb M. Cheeseman J. (2009). Gaussian 09 (revision D.01).

[B19] Genheden S. Ryde U. (2015). The MM/PBSA and MM/GBSA methods to estimate ligand-binding affinities. Expert Opin. Drug Discov. 10 (5), 449–461. 10.1517/17460441.2015.1032936 25835573 PMC4487606

[B20] Gigli M. Merlo M. Graw S. L. Barbati G. Rowland T. J. Slavov D. B. (2019). Genetic risk of arrhythmic phenotypes in patients with dilated cardiomyopathy. J. Am. Coll. Cardiol. 74 (11), 1480–1490. 10.1016/j.jacc.2019.06.072 31514951 PMC6750731

[B21] Harding D. Chong M. H. A. Lahoti N. Bigogno C. M. Prema R. Mohiddin S. A. (2023). Dilated cardiomyopathy and chronic cardiac inflammation: Pathogenesis, diagnosis and therapy. J. Intern Med. 293 (1), 23–47. 10.1111/joim.13556 36030368

[B22] Hsin K. Y. Ghosh S. Kitano H. (2013). Combining machine learning systems and multiple docking simulation packages to improve docking prediction reliability for network pharmacology. PLoS One 8 (12), e83922. 10.1371/journal.pone.0083922 24391846 PMC3877102

[B23] Huang J. Zhang J. Wang F. Zhang B. Tang X. (2022). Comprehensive analysis of cuproptosis-related genes in immune infiltration and diagnosis in ulcerative colitis. Front. Immunol. 13, 1008146. 10.3389/fimmu.2022.1008146 36389705 PMC9644813

[B24] Huang L. Shen R. Huang L. Yu J. Rong H. (2019). Association between serum copper and heart failure: A meta-analysis. Asia Pac J. Clin. Nutr. 28 (4), 761–769. 10.6133/apjcn.201912_28(4).0013 31826374

[B25] Huo S. Wang Q. Shi W. Peng L. Jiang Y. Zhu M. (2023). ATF3/SPI1/SLC31A1 signaling promotes cuproptosis induced by advanced glycosylation end products in diabetic myocardial injury. Int. J. Mol. Sci. 24 (2), 1667. 10.3390/ijms24021667 36675183 PMC9862315

[B26] Kanda T. Yokoyama T. Suzuki T. Murata K. (1992). Functional abnormalities of circulating natural killer cell subpopulations in patients with dilated cardiomyopathy. Tohoku J. Exp. Med. 168 (3), 529–537. 10.1620/tjem.168.529 1284796

[B27] Kim B. E. Turski M. L. Nose Y. Casad M. Rockman H. A. Thiele D. J. (2010). Cardiac copper deficiency activates a systemic signaling mechanism that communicates with the copper acquisition and storage organs. Cell Metab. 11 (5), 353–363. 10.1016/j.cmet.2010.04.003 20444417 PMC2901851

[B28] Korotkevich G. Sukhov V. Budin N. Shpak B. Artyomov M. N. Sergushichev A. (2021). Fast gene set enrichment analysis. bioRxiv [Preprint], 060012. New York: Cold Spring Harbor Laboratory, Cold Spring Harbor, 1802–1803. 10.1101/060012

[B29] Kräutler V. Gunsteren W. F. V. Hünenberger P. H. (2015). A fast SHAKE algorithm to solve distance constraint equations for small molecules in molecular dynamics simulations. J. Comput. Chem. 22 (5), 501–508.

[B30] Kühl U. Noutsias M. Schultheiss H. P. (1995). Immunohistochemistry in dilated cardiomyopathy. Eur. Heart J. 16, 100–106. 10.1093/eurheartj/16.suppl_o.100 8682073

[B31] Lai Y. Lin C. Lin X. Wu L. Zhao Y. Lin F. (2022). Identification and immunological characterization of cuproptosis-related molecular clusters in Alzheimer's disease. Front. Aging Neurosci. 14, 932676. 10.3389/fnagi.2022.932676 35966780 PMC9366224

[B32] Langfelder P. Horvath S. (2008). Wgcna: an R package for weighted correlation network analysis. BMC Bioinforma. 9, 559. 10.1186/1471-2105-9-559 PMC2631488 19114008

[B33] Larini L. Mannella R. Leporini D. (2007). Langevin stabilization of molecular-dynamics simulations of polymers by means of quasisymplectic algorithms. J. Chem. Phys. 126 (10), 104101. 10.1063/1.2464095 17362055

[B34] Lee J. Petris M. J. Thiele D. J. (2002). Characterization of mouse embryonic cells deficient in the ctr1 high affinity copper transporter. Identification of a Ctr1-independent copper transport system. J. Biol. Chem. 277 (43), 40253–40259. 10.1074/jbc.M208002200 12177073

[B35] Leek J. T. Johnson W. E. Parker H. S. Jaffe A. E. Storey J. D. (2012). The sva package for removing batch effects and other unwanted variation in high-throughput experiments. Bioinformatics 28 (6), 882–883. 10.1093/bioinformatics/bts034 22257669 PMC3307112

[B36] Lin X. Yang F. Zhou L. Yin P. Kong H. Xing W. (2012). A support vector machine-recursive feature elimination feature selection method based on artificial contrast variables and mutual information. J. Chromatogr. B Anal. Technol. Biomed. Life Sci. 910, 149–155. 10.1016/j.jchromb.2012.05.020 22682888

[B37] Liu Y. Dong Y. Wu X. Wang X. Niu J. (2022). Identification of immune microenvironment changes and the expression of immune-related genes in liver cirrhosis. Front. Immunol. 13, 918445. 10.3389/fimmu.2022.918445 35903097 PMC9315064

[B38] Maier J. A. Martinez C. Kasavajhala K. Wickstrom L. Hauser K. E. Simmerling C. (2015). ff14SB: Improving the accuracy of protein side chain and backbone parameters from ff99SB. J. Chem. Theory Comput. 11 (8), 3696–3713. 10.1021/acs.jctc.5b00255 26574453 PMC4821407

[B39] Mark P. Nilsson L. (2001). Structure and dynamics of the TIP3P, SPC, and SPC/E water models at 298 K. J. Phys. Chem. A 105 (43), 9954–9960. 10.1021/jp003020w

[B40] Ogunleye A. Wang Q. G. (2020). XGBoost model for chronic kidney disease diagnosis. IEEE/ACM Trans. Comput. Biol. Bioinform 17 (6), 2131–2140. 10.1109/tcbb.2019.2911071 30998478

[B41] Oluranti O. I. Adeyemo V. A. Achile E. O. Fatokun B. P. Ojo A. O. (2022). Rutin improves cardiac and erythrocyte membrane-bound ATPase activities in male rats exposed to cadmium chloride and lead acetate. Biol. Trace Elem. Res. 200 (3), 1181–1189. 10.1007/s12011-021-02711-4 33844168

[B42] Oluranti O. I. Alabi B. A. Michael O. S. Ojo A. O. Fatokun B. P. (2021). Rutin prevents cardiac oxidative stress and inflammation induced by bisphenol A and dibutyl phthalate exposure via NRF-2/NF-κB pathway. Life Sci. 284, 119878. 10.1016/j.lfs.2021.119878 34384828

[B43] Peng L. Ma M. Dong Y. Wu Q. An S. Cao M. (2022). Kuoxin Decoction promotes lymphangiogenesis in zebrafish and *in vitro* based on network analysis. Front. Pharmacol. 13, 915161. 10.3389/fphar.2022.915161 36105188 PMC9465995

[B44] Rastelli G. Del Rio A. Degliesposti G. Sgobba M. (2010). Fast and accurate predictions of binding free energies using MM-PBSA and MM-GBSA. J. Comput. Chem. 31 (4), 797–810. 10.1002/jcc.21372 19569205

[B45] Ritchie M. E. Phipson B. Wu D. Hu Y. Law C. W. Shi W. (2015). Limma powers differential expression analyses for RNA-sequencing and microarray studies. Nucleic Acids Res. 43 (7), e47. 10.1093/nar/gkv007 25605792 PMC4402510

[B46] Robin X. Turck N. Hainard A. Tiberti N. Lisacek F. Sanchez J. C. (2011). pROC: an open-source package for R and S+ to analyze and compare ROC curves. BMC Bioinforma. 12, 77. 10.1186/1471-2105-12-77 PMC3068975 21414208

[B47] Ruiz L. M. Libedinsky A. Elorza A. A. (2021). Role of copper on mitochondrial function and metabolism. Front. Mol. Biosci. 8, 711227. 10.3389/fmolb.2021.711227 34504870 PMC8421569

[B48] Sagui C. Darden T. A. (1999). Molecular dynamics simulations of biomolecules: Long-range electrostatic effects. Annu. Rev. Biophys. Biomol. Struct. 28, 155–179. 10.1146/annurev.biophys.28.1.155 10410799

[B49] Salomon-Ferrer R. Case D. A. Walker R. C. (2013). An overview of the Amber biomolecular simulation package. Wiley Interdiscip. Rev. Comput. Mol. Sci. 3 (2), 198–210. 10.1002/wcms.1121

[B50] Schultheiss H. P. Fairweather D. Caforio A. L. P. Escher F. Hershberger R. E. Lipshultz S. E. (2019). Dilated cardiomyopathy. Nat. Rev. Dis. Prim. 5 (1), 32. 10.1038/s41572-019-0084-1 31073128 PMC7096917

[B51] Schulz V. Basu S. Freibert S. A. Webert H. Boss L. Mühlenhoff U. (2022). Functional spectrum and specificity of mitochondrial ferredoxins FDX1 and FDX2. Nat. Chem. Biol. 19, 206–217. 10.1038/s41589-022-01159-4 36280795 PMC10873809

[B52] Schweigel-Röntgen M. (2014). The families of zinc (SLC30 and SLC39) and copper (SLC31) transporters. Curr. Top. Membr. 73, 321–355. 10.1016/b978-0-12-800223-0.00009-8 24745988

[B53] Stuart T. Butler A. Hoffman P. Hafemeister C. Papalexi E. Mauck W. M. 3rd (2019). Comprehensive integration of single-cell data. Cell 177 (7), 1888–1902.e21. 10.1016/j.cell.2019.05.031 31178118 PMC6687398

[B54] Sun R. Wang J. Zheng Y. Li X. Xie T. Li R. (2017). Traditional Chinese medicine baoxin decoction improves cardiac fibrosis of rats with dilated cardiomyopathy. Exp. Ther. Med. 13 (5), 1900–1906. 10.3892/etm.2017.4223 28565783 PMC5443197

[B55] Szklarczyk D. Gable A. L. Lyon D. Junge A. Wyder S. Huerta-Cepas J. (2019). STRING v11: Protein-protein association networks with increased coverage, supporting functional discovery in genome-wide experimental datasets. Nucleic Acids Res. 47 (D1), D607–d613. 10.1093/nar/gky1131 30476243 PMC6323986

[B56] Tsvetkov P. Coy S. Petrova B. Dreishpoon M. Verma A. Abdusamad M. (2022). Copper induces cell death by targeting lipoylated TCA cycle proteins. Science 375 (6586), 1254–1261. 10.1126/science.abf0529 35298263 PMC9273333

[B57] Walters A. M. Porter G. A. Jr. Brookes P. S. (2012). Mitochondria as a drug target in ischemic heart disease and cardiomyopathy. Circ. Res. 111 (9), 1222–1236. 10.1161/circresaha.112.265660 23065345 PMC3507431

[B58] Wang J. Wolf R. M. Caldwell J. W. Kollman P. A. Case D. A. (2004). Development and testing of a general AMBER force field. J. Comput. Chem. 25 (9), 1157–1174. 10.1002/jcc.20035 15116359

[B59] Wang K. Wang H. Wu J. Duan X. Liu X. Zhang D. (2021). Investigation on the efficiency of tonic Chinese herbal injections for treating dilated cardiomyopathy based on bayesian network meta-analysis. Evid. Based Complement. Altern. Med. 2021, 8838826. 10.1155/2021/8838826 PMC8035002 33868444

[B60] Wang X. Zhao Y. Strohmer D. F. Yang W. Xia Z. Yu C. (2022). The prognostic value of MicroRNAs associated with fatty acid metabolism in head and neck squamous cell carcinoma. Front. Genet. 13, 983672. 10.3389/fgene.2022.983672 36110217 PMC9468645

[B61] Wilkerson M. D. Hayes D. N. (2010). ConsensusClusterPlus: A class discovery tool with confidence assessments and item tracking. Bioinformatics 26 (12), 1572–1573. 10.1093/bioinformatics/btq170 20427518 PMC2881355

[B62] Wu W. Ziemann M. Huynh K. She G. Pang Z. D. Zhang Y. (2021). Activation of Hippo signaling pathway mediates mitochondria dysfunction and dilated cardiomyopathy in mice. Theranostics 11 (18), 8993–9008. 10.7150/thno.62302 34522223 PMC8419046

[B63] Xianchu L. Lan Z. Ming L. Yanzhi M. (2018). Protective effects of rutin on lipopolysaccharide-induced heart injury in mice. J. Toxicol. Sci. 43 (5), 329–337. 10.2131/jts.43.329 29743444

[B64] Yu L. Li Z. Dong X. Xue X. Liu Y. Xu S. (2018). Polydatin protects diabetic heart against ischemia-reperfusion injury via notch1/hes1-mediated activation of pten/akt signaling. Oxid. Med. Cell Longev. 2018, 2750695. 10.1155/2018/2750695 29636838 PMC5831600

[B65] Zhang Q. Fan M. Cao X. Geng H. Su Y. Wu C. (2022). Integrated bioinformatics algorithms and experimental validation to explore robust biomarkers and landscape of immune cell infiltration in dilated cardiomyopathy. Front. Cardiovasc Med. 9, 809470. 10.3389/fcvm.2022.809470 35433865 PMC9010553

[B66] Zhang Q. Tan Y. Zhang N. Yao F. (2015). Polydatin prevents angiotensin II-induced cardiac hypertrophy and myocardial superoxide generation. Exp. Biol. Med. (Maywood) 240 (10), 1352–1361. 10.1177/1535370214561958 25488910 PMC4935258

[B67] Zhao S. Chi H. Yang Q. Chen S. Wu C. Lai G. (2023). Identification and validation of neurotrophic factor-related gene signatures in glioblastoma and Parkinson's disease. Front. Immunol. 14, 1090040. 10.3389/fimmu.2023.1090040 36825022 PMC9941742

[B68] Zhu T. Wang M. Quan J. Du Z. Li Q. Xie Y. (2022). Identification and verification of feature biomarkers associated with immune cells in dilated cardiomyopathy by bioinformatics analysis. Front. Genet. 13, 874544. 10.3389/fgene.2022.874544 35646094 PMC9133742

[B69] Zhu Y. S. Li Y. L. Ju J. Q. Du F. Zang Y. P. Wang X. B. (2016). Oral Chinese herbal medicine for treatment of dilated cardiomyopathy: A systematic review and meta-analysis. Evid. Based Complement. Altern. Med. 2016, 1819794. 10.1155/2016/1819794 PMC5007314 27630730

