# Supplementary material for: Identification of cuproptosis-related biomarkers in dilated cardiomyopathy and potential therapeutic prediction of herbal medicines
Source: Front Mol Biosci. 2023 Apr 24;10:1154920. doi: 10.3389/fmolb.2023.1154920 (PMC10165005; doi:10.3389/fmolb.2023.1154920)
Supplement: Supplementary file 3 [file Table1.DOCX]

**Supplement Figure**

**
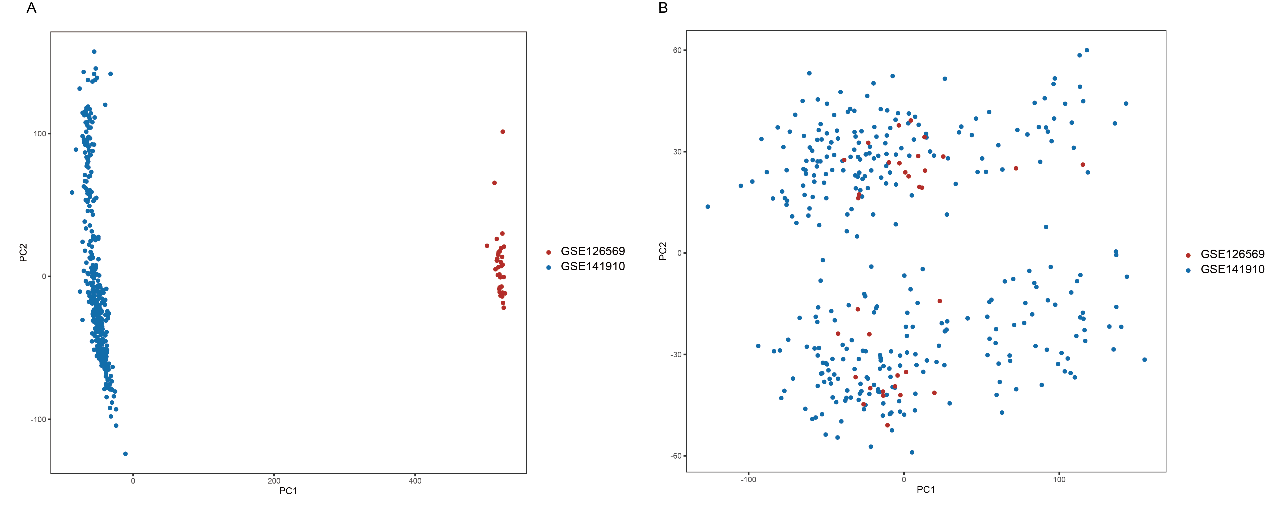
**

**Figure 1 GSE141910 and GSE126569 dataset batch effects.**(A) Batch effect GSE141910 and GSE126569 datasets evaluated by PCA. (B) PCA showed the GSE141910 and GSE126569 datasets after the batch effect was removed.
